# Nestedness Maximization in Complex Networks through the Fitness-Complexity Algorithm

**DOI:** 10.3390/e20100768

**Published:** 2018-10-08

**Authors:** Jian-Hong Lin, Claudio Juan Tessone, Manuel Sebastian Mariani

**Affiliations:** 1 URPP Social Networks, University of Zurich, CH-8050 Zurich, Switzerland; 2 Institute of Fundamental and Frontier Science, University of Electronic Science and Technology of China, Chengdu 610054, China

**Keywords:** economic fitness, fitness-complexity, genetic algorithms, nestedness temperature, ecological networks

## Abstract

Nestedness refers to the structural property of complex networks that the neighborhood of a given node is a subset of the neighborhoods of better-connected nodes. Following the seminal work by Patterson and Atmar (1986), ecologists have been long interested in revealing the configuration of maximal nestedness of spatial and interaction matrices of ecological communities. In ecology, the BINMATNEST genetic algorithm can be considered as the state-of-the-art approach for this task. On the other hand, the fitness-complexity ranking algorithm has been recently introduced in the economic complexity literature with the original goal to rank countries and products in World Trade export networks. Here, by bringing together quantitative methods from ecology and economic complexity, we show that the fitness-complexity algorithm is highly effective in the nestedness maximization task. More specifically, it generates matrices that are more nested than the optimal ones by BINMATNEST for 61.27% of the analyzed mutualistic networks. Our findings on ecological and World Trade data suggest that beyond its applications in economic complexity, the fitness-complexity algorithm has the potential to become a standard tool in nestedness analysis.

## 1. Introduction

**Figure 1:**
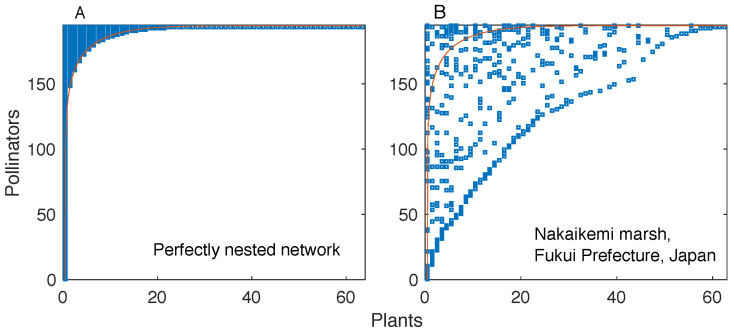
An illustration of the interaction matrix of a perfectly nested network as compared to the interaction matrix of a non-nested network (Nakaikemi marsh pollination network) composed of the same number of nodes and links. In a perfectly nested network (**left panel**), one can define a line (marked in red) that perfectly partitions the matrix into a filled region (i.e., the region above the line) and an empty region (i.e., the region below the line). The same feature does not hold for a non-nested network (**right panel**).

Network representations of complex interacting systems provide simple and powerful frameworks to characterize the topology of interactions and understand its impact on the emergence of collective phenomena [1,2]. Some topological properties are found in a wide variety of real networks, which has led scholars to investigate possible interaction mechanisms behind their emergence. An example is the heavy-tailed distribution of the number of links per node (degree); its ubiquity has motivated the study of various network growth mechanisms that can generate networks with that property [2]. First conceived [3] and measured [4,5] in biogeographic studies, *nestedness* [6] is one of such pervasive properties. In a perfectly nested bipartite network, the interaction partners of a given node are also partners of more generalist nodes. This property results in a “triangular” shape of the network’s interaction matrix (i.e., the binary matrix whose elements denote the presence or absence of a link, see Figure 1).

While perfectly nested networks are unambiguously defined, they are also rarely found in real systems. However, many real networks exhibit a high degree of nestedness. The degree of nestedness of a bipartite network has not been uniquely defined in the literature [6]. In the widely adopted definition by Atmar and Patterson [5], which is the one we consider here, a network is highly nested if the rows and columns of its interaction matrix can be ordered in such a way that one can find a line that separates almost perfectly the filled and empty regions of the matrix. It is essential to notice that this definition involves a reordering of the interaction matrix’s rows and columns; alternative definitions of nestedness [7,8] (not considered here) do not involve any matrix reordering.

Based on various metrics and definitions, nestedness has indeed been found in systems as diverse as spatial patterns of species distribution [4,6], mutualistic plant-animal networks [9], manufacturer-contractor networks [10,11], country-product export networks [12,13], spatial patterns of firm distribution [12,14], among others. The ubiquity of the pattern has naturally led scholars to investigate how nestedness relates to other network properties [15,16,17], which mechanisms can possibly explain its emergence in ecological [18,19,20] and socio-economic [10,21,21] networks, and its implications for the stability and feasibility of ecological systems [22,23].

One of the most popular algorithms to quantify the degree of nestedness of a given network is the *Nestedness Temperature Calculator* [5]. Introduced by Atmar and Patterson in 1993 [5], the algorithm first determines a line of perfect nestedness by defining a perfectly nested interaction matrix with the same number of links as the original matrix. Then, it seeks to find the ranking of rows and columns that minimizes the average distance (“temperature” [5]) of observed “unexpected” matrix elements from the line of perfect nestedness; the unexpected matrix elements are those that are different from the corresponding ones in a perfectly nested matrix with the same number of links as the original matrix. Lower temperatures correspond to more nested topologies.

While the original Nestedness Temperature Calculator (NTC) by Atmar and Patterson [5] has been widely used in ecology [6], it exhibits some shortcomings that have been later overcome by the BINMATNEST algorithm [24]. BINMATNEST minimizes nestedness temperature through a genetic algorithm that confers higher chance to reproduce upon lower-temperature orderings [24]. The optimal matrices by BINMATNEST exhibit substantially lower temperature than those ranked by the NTC [24], which is why BINMATNEST can be considered as the state-of-the-art approach for nestedness temperature minimization in ecology.

Here, we explore an alternative approach to nestedness temperature minimization inspired by the recent Economic Complexity literature [25,26]. Originally introduced to rank countries and products in the country-product export network [25], the fitness-complexity algorithm ranks the countries and products in such a way that the resulting incidence matrix exhibits a (typically imperfect) “triangular” shape [25,26,27,28]. In World Trade, this suggests that the most competitive countries tend to diversify their export baskets, whereas the most sophisticated products can be only fabricated by the most competitive countries [25,26]. The country score produced by the algorithm, referred to as country fitness, is positively correlated with country GDP per capita [25,26]. Importantly, deviations from the linear-regressed trend are highly informative about the future economic development of the country [29,30], resulting in GDP predictions often more accurate than those by the International Monetary Fund [31,32].

The fact that matrices sorted according to the fitness-complexity algorithm exhibit a neater “triangular” shape than those sorted by degree [27] suggests that the algorithm might be competitive with algorithms typically adopted in ecology for nestedness temperature minimization [33]. The main goal of this article is to extensively compare the fitness-complexity algorithm and BINMATNEST according to their ability to minimize nestedness temperature. To this end, we analyze 142 mutualistic networks from http://www.web-of-life.es/ and 14 years of World Trade country-product networks from https://atlas.media.mit.edu/ en/resources/data/. We compare the nestedness temperature of the matrices as ranked by BINMATNEST with those of the same matrices as ranked by the fitness-complexity algorithm.

We find that the fitness-complexity algorithm generates sorted matrices that exhibit a lower temperature than the optimal matrices by BINMATNEST for the 61.27% of the analyzed ecological networks. The only matrices where BINMATNEST outperforms substantially the fitness-complexity algorithm are low-size and high-density ones. The FCA is marginally outperformed by BINMATNEST for World Trade networks which exhibit higher density than mutualistic networks of similar size. Our findings suggest that while originally introduced as a ranking algorithm in economic production networks, the fitness-complexity algorithm has the potential to become a standard tool for nestedness detection in complex networks.

## 2. Materials and Methods

This paper focuses on binary bipartite networks. We label row-nodes (countries/pollinators) and column-nodes (products/plants) through Latin (i∈{1,…,N}) and Greek (α∈{1,…,M}) letters, respectively. The total number of row-nodes and column-nodes is denoted as *N* and *M*, respectively, whereas the total number of links is denoted as *L*. The N×M network’s *incidence matrix* [1] is denoted as B: its element $B_{i\alpha }$ is equal to one (“filled” element) if link (i,α) is observed, zero (“empty” element) otherwise. We refer to the incidence matrix of mutualistic networks as *interaction matrix* [9]. The density Φ of the network is defined as Φ=L/(MN).

### 2.1. Nestedness Temperature Minimization (NTM) Problem

Nestedness temperature is determined through three steps: determination of the line of perfect nestedness, node ranking, and temperature calculation. We provide below the details of the three steps, and state the NTM problem.

First, to compute the nestedness temperature of a given matrix, one needs to determine its *line of perfect nestedness*. In this work, we use the definition provided by Rodríguez-Gironés and Santamaría [24] which overcomes some of the shortcomings of the original geometrical construction by Atmar and Patterson [5]. By rescaling the row and columns labels in such a way that they range from 0 to 1, the line of perfect nestedness is determined through the following shape function [24]

(1) $$f\left(x;p\right)=\frac{0.5}{N}+\frac{N-1}{N}{\left(1-{\left(1-\frac{M\;x-0.5}{M-1}\right)}^{p}\right)}^{1/p}.$$

This function depends on a single parameter, *p*, which is determined by imposing that the area above the curve in the interval (0,1) equals the fill of the matrix Φ.

Second, matrix temperature depends on the order of rows and columns. The *nestedness temperature minimization (NTM) problem* (or, equivalently, the *nestedness maximization problem*) consists in determining the ranking of rows and columns that produces a ranked matrix of minimal temperature *T* (defined below). The output of this step is, therefore, a pair of rankings, one for rows and one for columns. Equivalently, we can say that the output of the ranking is a *ranked matrix*. Due to the large number of possible permutations of rows and columns, a combinatorial search is infeasible [24], which has motivated ecologists to search for fast ranking methods [5,24,34]. The main goal of this paper is to compare two alternative ranking algorithms, the one adopted by BINMATNEST (details in Section 2.2) and the fitness-complexity algorithm (details in Section 2.3).

Third, for a given network and a given ranking of its row-nodes and column-nodes, one calculates nestedness temperature *T* as follows. The unexpected elements of the ranked matrix are the the empty elements above and the filled elements below the line of perfect nestedness (as determined through Equation (1)). We denote by U the set of unexpected elements. For each unexpected element (i,α), one draws a straight line of slope -1 in the interaction matrix (after having normalized to one the column and row labels, as described above). On this line, one compute the distance $d_{i\alpha }$ of unexpected element (i,α) from the line of perfect nestedness, and the distance $D_{i\alpha }$ between the intersection points of this line with the *x*-axis and *y*-axis (see Figure 1 in [24] for an illustration). The total unexpectedness *U* of the ranked matrix is given by [5,24]

(2) $$U=\frac{1}{N\;M}\sum_{(i,\alpha )\in U}{\left(\frac{d_{i\alpha }}{D_{i\alpha }}\right)}^{2}.$$

Matrix temperature is defined as $T=100\;U/U_{max}$, where $U_{max}=0.04145$ [5,24]. A perfectly nested matrix has zero temperature (“perfect order” [5]), whereas random, noisy matrices have large temperature.

We stress that the key point in our analysis is that the calculation of nestedness temperature *T* requires a ranked matrix as input: different rankings of rows and columns lead to different matrix temperatures. This allows us to compare different ranking algorithms with respect to the nestedness temperature they produce. We expect the rankings by effective algorithms for NTM to produce ranked matrices that exhibit lower temperature than the ranked matrices by other algorithms.

### 2.2. Genetic Algorithm Approach: BINMATNEST (BIN)

The BINMATNEST algorithm [24] adopts a genetic-algorithm approach [35] to the NTM problem. As the computational steps of the ranking algorithm are detailed in [24], we only discuss here the main ideas behind the algorithm. The goal is to find a “solution” to the NTM problem, i.e., the minimal-temperature ranking of the nodes. The algorithm starts with a set of candidate solutions (“chromosomes” in the genetic-algorithm language [35]); among these solutions, the rankings by degree and by the Nestedness Temperature Calculator by Atmar and Patterson [5]. In each generation, the algorithm considers a well-performing solution, and it generates an “offspring” solution o by probabilistically combining elements of the well-performing solution w with elements of a randomly selected “partner” solution p.

More specifically, let us consider the ranking of the row-nodes. Given a well-performing solution $w=\{w_{1},\ldots ,w_{N}\}$ and a partner solution $p=\{p_{1},\ldots ,p_{N}\}$, the each element of the offspring solution is given by the corresponding element of w with probability 1/2; otherwise, it is determined by the following steps:

- We randomly select an integer *k* between 1 and *N*.
- We set $o_{i}=w_{i}$ for i∈{1,…,k}.
- We set $o_{i}=p_{i}$ for i∈{k+1,…,N}, if and only if $p_{i}\notin \left\{w_{1},\ldots ,w_{k}\right\}$.
- If $p_{i}\in \left\{w_{1},\ldots ,w_{k}\right\}$, we assign one of the ranking positions that have not yet appeared in o to $0_{i}$.

One applies the same steps to the ranking of the column-nodes.Besides, after these steps are performed, the offspring solution can undergo a mutation with a given probability (set to 0.1 in [24]). If the mutation happens, in the case of row-nodes, one extracts uniformly at random two integers $k_{1},k_{2}\in \left\{1,\ldots ,N\right\}$ ($k_{1}<k_{2}$), and cyclically permutes the elements $\left\{o_{k_{1}},\ldots ,o_{k_{2}}\right\}$. The process described above is iterated for a given number of generations, and the minimal-temperature solution is eventually selected to determine the network nestedness temperature.

The output of the BINMATNEST algorithm is therefore a ranking of the rows and columns that minimizes nestedness temperature *T*. Importantly, the optimal rankings by BINMATNEST lead to temperature values that are substantially lower than those determined by the widely used Nestedness Temperature Calculator [5]; see Figs. 4–5 in [24], for example. Based on those results, BINMATNEST can be considered as the state-of-the-art approach for NTM in ecological networks. In this paper, we implement the BINMATNEST algorithm by using the function nestedrank (https://www.rdocumentation.org/packages/bipartite/versions/2.11/topics/nestedrank) from the R package bipartite with argument method = “binmatnest”. This function gives as output the ranking of row-nodes and column-nodes by the BINMATNEST algorithm.

### 2.3. Non-Linear Iterative Algorithms: Fitness-Complexity Algorithm (FCA)

Originally introduced to rank countries and products in the bipartite country-product export network [25], the fitness-complexity algorithm has been applied to diverse systems including ecological mutualistic networks [33], knowledge production networks [36], food production networks [37]. In its formulation for countries and products [25], the algorithm aims to find a vector of “fitness” scores $F=\{F_{i}\}$ for countries and “complexity” scores $Q=\{Q_{\alpha }\}$ for products, respectively. The algorithm starts from a uniform initial condition [25]

(3) $$\begin{array}{cc} F_{i}^{\left(0\right)} & =1,\\ Q_{\alpha }^{\left(0\right)} & =1, \end{array}$$

and it subsequently refines the fitness and complexity scores according to the following non-linear iterative equations:

(4) $$\begin{array}{cc} {\tilde{F}}_{i}^{\left(n\right)} & =\sum_{\alpha }B_{i\alpha }Q_{\alpha }^{\left(n-1\right)},\\ {\tilde{Q}}_{\alpha }^{\left(n\right)} & =\frac{1}{\sum_{i}B_{i\alpha }/F_{i}^{\left(n-1\right)}}. \end{array}$$

After each iterative step, the scores are normalized by their mean:

(5) $$\begin{array}{cc} F_{i}^{\left(n\right)} & ={\tilde{F}}_{i}^{\left(n\right)}/\langle {\tilde{F}}_{i}^{\left(n\right)}\rangle ,\\ Q_{\alpha }^{\left(n\right)} & ={\tilde{Q}}_{\alpha }^{\left(n\right)}/\langle {\tilde{Q}}_{\alpha }^{\left(n\right)}\rangle . \end{array}$$

Differently from widely used spectral ranking algorithms (see [30] for a review), the second line of Equation (4) is markedly non-linear. Such non-linearity is motivated by economic-complexity considerations. Empirical evidence indicates indeed that competitive countries tend to diversify their export baskets, which makes it reasonable to quantify the score of a given country as the sum over the scores of its exported products. At the same time, the fact that a product is exported by many countries (in particular, developing countries) suggests that the product might require few productive capabilities to be made and it is unlikely to be a sophisticated one. This motivates the non-linear dependence of product score ${\tilde{Q}}_{\alpha }^{\left(n\right)}$ on country score $F_{i}^{\left(n-1\right)}$: ${\tilde{Q}}_{\alpha }^{\left(n\right)}$ is heavily penalized if α is exported by a low-fitness country.

Do the iterations above converge to a unique fixed point? Scholars have found that while the answer is positive, the scores of several nodes can potentially converge to a zero value, which reduces the discriminative power of the ranking based on the fixed point of the map [38]. Besides, this convergence to zero tends to be relatively slow, and it strongly depends on the density and shape of the incidence matrix [28,38]. To prevent this potential issue, we adopt a convergence criterion based on ranking: we stop the iterations at step $n^{*}$ if and only if the ranking of countries and products at step $n^{*}$ is almost exactly the same as the ranking at step $n^{*}+\Delta n$, i.e., if few ranking variations occurred in the subsequent Δn steps. In practice, the stopping iteration $n^{*}$ is defined as the smallest iteration such that both Spearman’s correlation coefficients $\rho (F^{\left(n^{*}\right)},F^{\left(n^{*}+\Delta n\right)})$ and $\rho (Q^{\left(n^{*}\right)},Q^{\left(n^{*}+\Delta n\right)})$ are larger than $1-10^{-3}$. Unless otherwise stated, the results presented in this manuscript refer to Δn=10 – the criterion allows us to stop the algorithm after a finite number of iteration for all the analyzed networks. We find that results for Δn=20 and Δn=30 are in qualitative agreement with those obtained with Δn=10; the same holds for results obtained by running a fixed number $n^{*}=100$ of iterations of the FCA – details are provided in the Results section.

While we formulated the algorithm for the country-product network, the algorithm can be applied to any bipartite network by replacing “countries” with the system’s row-nodes (e.g., animals in mutualistic networks [33]) and “products” with the system’s column-nodes (e.g., plants). In this paper, we apply it not only to the country-product network, but also to mutualistic networks: the fitness score of animal and plant species represents their importance and vulnerability, respectively [33].

## 3. Results

### 3.1. Mutualistic Networks

**Figure 2:**
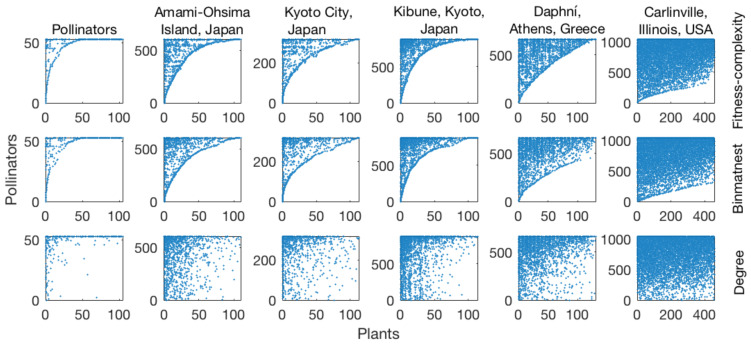
Six empirical mutualistic matrices of different density packed according to three different methods: fitness-complexity algorithm (**top row**), BINMATNEST (intermediate row), and degree (**bottom row**). The matrices ranked by fitness-complexity and BINMATNEST are significantly more nested than those ranked by degree.

We analyzed the 142 pollination networks provided by The Web of Life (www.web-of-life.es) project. The species are plants (rows) and pollinators (columns) and the type of interaction is Pollination. The main goal of our paper is to compare the FCA and the BINMATNEST algorithm with respect to their performance in the NTM problem. Figure 2 shows that qualitatively, the matrices produced by the fitness-complexity algorithm are substantially more nested than those produced by ranking the nodes by degree, and their nestedness might be comparable or even larger than that of the matrices ranked by BINMATNEST.

The reason why the FCA produces highly nested structures is that the score of a plant/product is mostly determined by the least-fit pollinator/country (Such dependence can be even sharpened by replacing $1/F^{\left(n\right)}$ with ${\left(1/F^{\left(n\right)}\right)}^{\gamma }$ (with γ>0) in the dependence of the complexity score on fitness score (second line of Equation (4)) [27,38], or by defining the complexity of a product directly as the minimum fitness of its interaction partners [28]. However, we do not explore these possibilities here.): a plant/product that is pollinated/produced by a generalist pollinator/country — i.e., many pollinators/countries can pollinate/produce it, is heavily penalized and achieves a low complexity score *Q*; whereas a plant/product that is only pollinated/produced by specialist pollinator/country, i.e., few pollinators/countries can pollinate/produce it — attains a high complexity score. Hence, when sorting plants/products and pollinators/countries by the FCA, the plants/products are essentially ranked by the degree of generalization of their least-fit pollinators/exporters, which naturally results in a nested structure.

**Figure 3:**
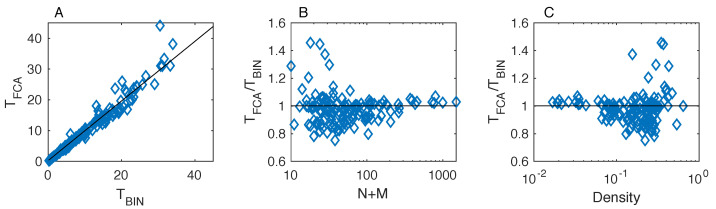
Results on mutualistic networks: a comparison of the nestedness temperature $T_{FC}$ of the matrices ranked by the FCA with the nestedness temperature $T_{BIN}$ of the optimal matrices found by the BINMATNEST genetic algorithm. The two temperatures are positively correlated (**panel A**), yet the temperature measured by the fitness-complexity algorithm is lower than that by BINMATNEST for the majority of analyzed networks. The only networks where BINMATNEST produces a substantially lower temperature ($T_{FCA}/T_{BIN}>1$) are characterized by small size N+M (**panel B**) and high density Φ (**panel** C).

We now proceed in a more quantitative fashion by comparing, for all the analyzed empirical networks, the temperature values produced by the FCA with those by BINMATNEST. To do this, for the rankings determined by both methods, we determine the corresponding matrix temperature *T* according to Equation (2). We find that while the temperature values achieved by the two methods are positively correlated (Figure 3A), the temperature $T_{FCA}$ by the FCA is lower than the temperature $T_{BIN}$ by BINMATNEST for 61.27% of the networks. This result is stable with respect to variations in the convergence criterion adopted for the FCA (This result was obtained with Δn=10. The fraction of datasets where $T_{FCA}<T_{BIN}$ is equal to 61.97% and 61.97% for Δn=20 and Δn=30, respectively. Besides, the same fraction was equal to 62.68% when using a fixed number $n^{*}=100$ of iterations for all the networks. We conclude that the fraction of datasets where $T_{FCA}<T_{BIN}$ is not substantially affected by the adopted convergence criterion for the FCA.).

The only matrices where the FCA is substantially outperformed by BINMATNEST are characterized by small size (Figure 3B) and high density (Figure 3C), yet these two properties seem necessary but not sufficient for BINMATNEST to outperform the FCA. Interestingly, among matrices that are found to be “colder” by the FCA, the lowest $T_{FCA}/T_{BIN}$ ratio ($T_{FCA}/T_{BIN}=0.75$) was observed in the M_PL_060_13 network (N=31,M=7,L=48); in this dataset, $T_{BIN}=10.15$ whereas $T_{FCA}=7.64$. By contrast, among matrices that are found to be “colder” by BINMATNEST, the highest $T_{FCA}/T_{BIN}$ ratio ($T_{FCA}/T_{BIN}=1.46$) was observed in the M_PL_042 network (N=6,M=12,L=18).

**Figure 4:**
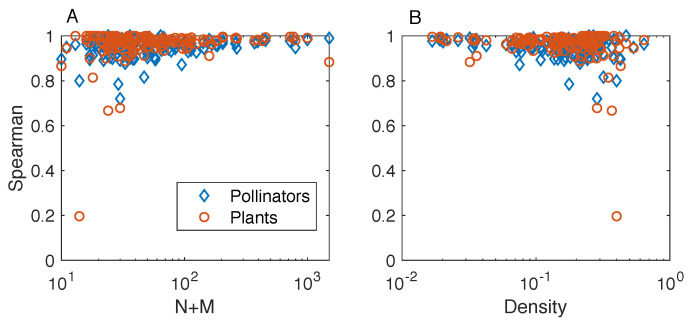
Results on mutualistic networks: Spearman’s rank correlation coefficient ρ between the rankings by BINMATNEST and the fitness-complexity algorithm, for the rankings of pollinators (rhombuses) and plants (circles). Panels A and B represent ρ as a function of size N+M and density Φ, respectively. The two methods produce highly correlated rankings: the networks where we observe the lowest values of correlation are the small (panel A) and high-density ones (panel B).

To deepen our understanding of the relation between the rankings by the FCA and BINMATNEST, we study their correlation and how such correlation depends on network properties. The Spearman’s correlation coefficient [39] between the rankings by the two methods is positive and relatively high for both plants and pollinators (Figure 4). Yet, as we have seen in Figure 3, discrepancies between the two rankings point to a better ability of the FCA to “pack” the matrix in such a way that it displays a nested structure. The networks where we observe the largest discrepancies between the rankings by BINMATNEST and the FCA are the small and high-density ones – for example, the minimal observed correlation for the rankings of pollinators is ρ=0.20, observed for one of the smallest networks [M_PL_069_02 which has N=4,M=10,L=16]. All the other Spearman’s coefficient values are above 0.67.

### 3.2. Country-Product Networks

We analyzed 14 years of World Trade data obtained from https://atlas.media.mit.edu/en/resources/data/. The raw data include information on which country exported which products to which countries, and the volume (measured in US dollars) of each trade relation. For each country-product pair (i,α), we denote by $w_{i\alpha }$ the volume of product α exported by country *i*. In line with the Economic Complexity literature [25,26,40], we construct a binary country-product network by only keeping the links between those country-product pairs such that $R_{i\alpha }\geq 1$, where $R_{i\alpha }:=w_{i\alpha }/\langle w_{i\alpha }\rangle$ is referred to as *revealed comparative advantage* [25], $\langle w_{i\alpha }\rangle =w_{i}\;w_{\alpha }/W$ denotes the expected weight based on the total export volume $w_{i}:=\sum_{\beta }w_{i\beta }$ of country *i*, the total export volume $w_{\alpha }:=\sum_{j}w_{j\alpha }$ of product α, and the total export volume $W=\sum_{j\beta }w_{j\beta }$ in the system. In other words, a given country *i* is connected to a given product α in the bipartite country-product network if and only if the export volume $w_{i\alpha }$ exceeds the expected export volume. Based on this assumption, we construct 14 binary networks corresponding to the 2001–2014 period.

**Figure 5:**
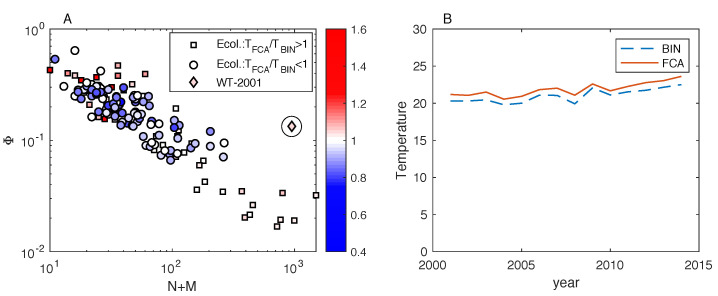
Results on mutualistic and World Trade networks. In (**panel A**), each dot represents a network in the size-density plane; the dots’ shape and color depend on the $T_{FCA}/T_{BIN}$ ratio, in such a way that mutualistic networks with a ratio larger or smaller than one are represented by red squares or blue circles, respectively. This illustration confirms that the mutualistic networks where $T_{FCA}$ is substantially larger than $T_{BIN}$ are characterized by small size and high density. The World Trade network from 2001 (represented by the circled rhombus) exhibits relatively high density compared to mutualistic networks of comparable size; World Trade networks from other years (2002–2014) exhibit a similar size and density as the one from 2001, and they are not shown here. (**Panel B**) shows that the temperature $T_{BIN}$ by BINMATNEST is marginally smaller than the temperature $T_{FCA}$ by the FCA for all the analyzed years of World Trade, and the temperature values do not exhibit wide fluctuations over time.

Figure 5 compares the temperature by the FCA and BINMATNEST in the size-density plane, for all the analyzed mutualistic networks and the World Trade networks. The figure reveals that compared to the mutualistic networks analyzed above, the obtained country-product networks turn out to have a similar size as the largest mutualistic networks, but substantially larger density (see Figure 5A). For all the analyzed World Trade networks, the temperature by BINMATNEST is marginally smaller than the one by the FCA, and both temperatures are stable over the years (see Figure 5B): the average of $T_{FCA}/T_{BIN}$ over the 14 analyzed years is equal to 1.04.

## 4. Discussion

We showed that the fitness-complexity ranking algorithm [25] is a highly effective method to “pack” the incidence matrix of a given bipartite network in order to maximize its nestedness. In particular, an extensive comparison with BINMATNEST, the state-of-the-art nestedness maximization method in ecology, revealed that the FCA produces ranked matrices with temperature values substantially lower than those of the optimal matrices by BINMATNEST for the majority of analyzed datasets. Small-size and high-density ecological matrices are those where the rankings by the two methods differ the most, and where BINMATNEST has a chance to produce matrices of significantly smaller temperature than those ranked by the fitness-complexity algorithm.

Importantly, the Nestedness Temperature Minimization problem is not only a theoretical one, but it has also implications for the important problem of forecasting of the secondary effects of species’ extinctions [33]. More specifically, recent works [27,33] have pointed out that the rankings of active and passive species (countries and products, in World Trade analysis [27]) that result in the most packed matrices are also those that best reproduce the rankings of the nodes according to their structural importance and vulnerability (as determined by numerical simulations of ranking-based targeted attacks to the network). Maximizing nestedness is therefore highly informative on the structural importance of active species and vulnerability of passive species.

Finally, recent literature has reinterpreted nestedness as a mesoscopic property instead of a macroscopic one [17,41,42]. This means that nestedness can be interpreted not as a hierarchical organization of interactions between all pairs of nodes (as in Figure 1), but as a property of subcomponents of the network. While our results show that the fitness-complexity algorithm can be used as a nestedness detection tool, whether it can be exploited (and arguably, generalized) to detect network compartments that exhibit an internal nested topology remains an intriguing open question.

## Abbreviations

The following abbreviations are used in this manuscript:

BIN	BINMATNEST algorithm
NTC	Nestedness Temperature Calculator
NTM	Nestedness Temperature Minimization
FCA	Fitness-Complexity algorithm
